# Crystal structure of alluaudite-type Na_4_Co(MoO_4_)_3_

**DOI:** 10.1107/S1600536814016729

**Published:** 2014-08-01

**Authors:** Rawia Nasri, Noura Fakhar Bourguiba, Mohamed Faouzi Zid, Ahmed Driss

**Affiliations:** a Laboratoire de Matériaux et Cristallochimie, Faculté des Sciences de Tunis, Université de Tunis El Manar, 2092 El Manar Tunis, Tunisia

**Keywords:** crystal structure, X-ray diffraction, molybdate, alluaudite

## Abstract

The title compound, tetra­sodium cobalt(II) tris­[molyb­date(IV)], was prepared by solid-state reactions. The structure is isotypic with Na_3_In_2_(AsO_4_)_3_ and Na_3_In_2_(PO_4_)_3_. The main structural feature is the presence of infinite chains of edge-sharing *X*_2_O_10_ (*X* = Co/Na) dimers, which are linked by MoO_4_ tetra­hedra, forming a three-dimensional framework enclosing two types of hexa­gonal tunnels in which Na^+^ cations reside. In this alluaudite structure, Co and Na atoms are located at the same general site with occupancies of 0.503 (5) and 0.497 (6), respectively. The other three Na and one of the two Mo atoms lie on special positions (site symmetries 2, -1, 2 and 2, respectively). The structure is compared with similar structures and other members of alluaudite family.

## Related literature   

For the bond-valance-sum method, see: Brown & Altermatt (1985 ▶). For related structures, see: Chaalia *et al.* (2012 ▶); Engel *et al.* (2005 ▶); Frigui *et al.* (2012 ▶); Hatert (2006 ▶); Hidouri *et al.* (2006 ▶); Kabbour *et al.* (2011 ▶); Kelvtsova *et al.* (1991 ▶); Lii & Ye (1997 ▶); Marzouki *et al.* (2013 ▶); Mikhailova *et al.* (2010 ▶); Moore (1971 ▶); Namsaraeva *et al.* (2011 ▶); Solodovnikov *et al.* (1988 ▶); Yakubovich *et al.* (2005 ▶).

## Experimental   

### Crystal data  

Na_4_Co(MoO_4_)_3_
*M* *_r_* = 630.71
Monoclinic, 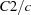

*a* = 12.8770 (8) Å
*b* = 13.4384 (9) Å
*c* = 7.1292 (7) Å
β = 112.072 (6)°
*V* = 1143.27 (15) Å^3^
*Z* = 4
Mo *K*α radiation
μ = 4.85 mm^−1^
*T* = 298 K
0.84 × 0.28 × 0.22 mm

### Data collection  

Enraf–Nonius CAD-4 diffractometer
Absorption correction: ψ scan (North *et al.*, 1968 ▶) *T*_min_ = 0.214, *T*_max_ = 0.344
2898 measured reflections
1242 independent reflections
1156 reflections with *I* > 2σ(*I*)
*R*_int_ = 0.036
2 standard reflections every 120 min intensity decay: 1.4%

### Refinement  

*R*[*F* ^2^ > 2σ(*F* ^2^)] = 0.025
*wR*(*F* ^2^) = 0.065
*S* = 1.12
1242 reflections
95 parameters
Δρ_max_ = 1.14 e Å^−3^
Δρ_min_ = −0.81 e Å^−3^

### 

Data collection: *CAD-4 EXPRESS* (Duisenberg, 1992 ▶; Macíček & Yordanov, 1992 ▶); cell refinement: *CAD-4 EXPRESS*; data reduction: *XCAD4* (Harms & Wocadlo, 1995 ▶); program(s) used to solve structure: *SHELXS97* (Sheldrick, 2008 ▶); program(s) used to refine structure: *SHELXL97* (Sheldrick, 2008 ▶); molecular graphics: *DIAMOND* (Brandenburg & Putz, 1999 ▶); software used to prepare material for publication: *WinGX* (Farrugia, 2012 ▶).

## Supplementary Material

Crystal structure: contains datablock(s) I. DOI: 10.1107/S1600536814016729/br2240sup1.cif

Structure factors: contains datablock(s) I. DOI: 10.1107/S1600536814016729/br2240Isup2.hkl

**Fig. 1.**
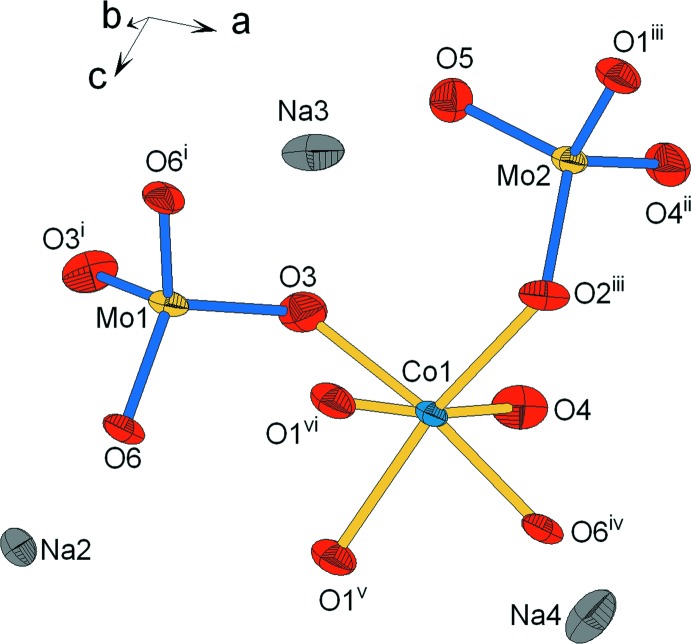
Unité asymétrique dans Na4Co(MoO4)3. Les éllipsoïdes ont été définis avec 50% de probabilité. [Code de symétrie]: (i) -x,y,-z + 1/2; (ii) x,y + 1,z; (iii) x,-y + 1,z - 1/2; (iv) -x + 1/2,-y + 1/2,-z + 1; (v) x,-y + 1,z + 1/2; (vi) -x + 1/2,y - 1/2,-z + 1/2.

**Fig. 2.**
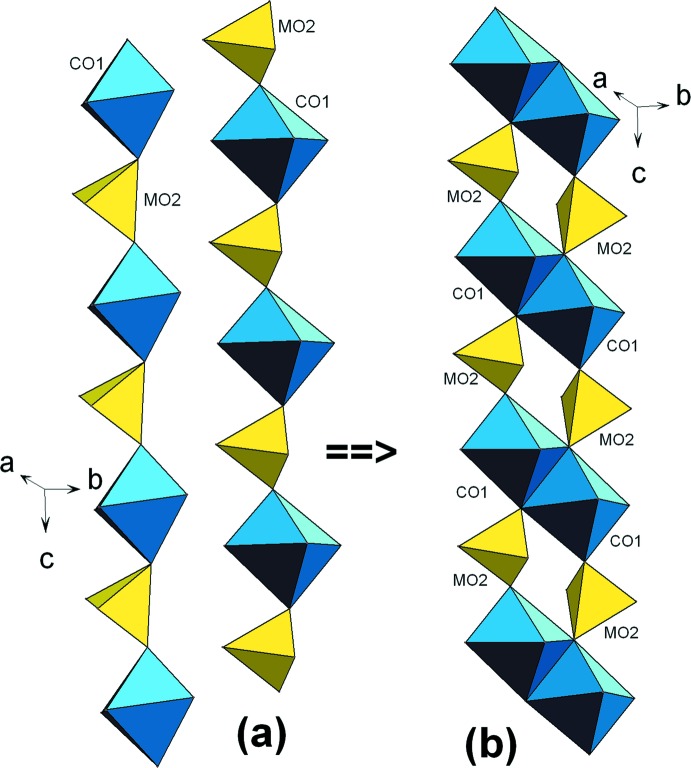
Représentation: (a) des chaînes classiques CoMoO8, (b) des rubans de type Co2Mo2O14.

Click here for additional data file.asym 4 4 3 Code de symétrie x y z x y z x y z x y z x y z x y z . DOI: 10.1107/S1600536814016729/br2240fig1.tif
Unité *asym*étrique dans Na_4_Co(MoO_4_)_3_. Les éllipsoïdes ont été définis avec 50% de probabilité. [*Code de symétrie*]: (i) −*x*,*y*,-*z* + 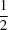; (ii) *x*,*y* + 1,*z*; (iii) *x*,-*y* + 1,*z* − 1/2; (iv) −*x* + 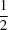,-*y* + 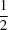,-*z* + 1; (v) *x*,-*y* + 1,*z* + 1/2; (vi) −*x* + 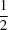,*y* − 1/2,-*z* + 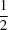.

a 8 b 2 2 14 . DOI: 10.1107/S1600536814016729/br2240fig2.tif
Représentation: (*a*) des chaînes classiques CoMoO_8_, (*b*) des rubans de type Co_2_Mo_2_O_14_.

**Fig. 3.**
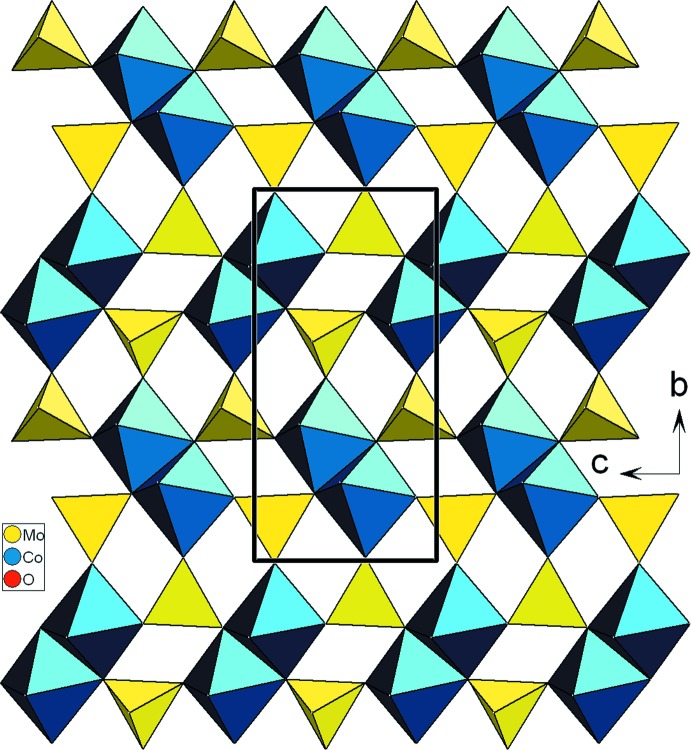
Représentation des couches disposées parallèlement au plan (100).

. DOI: 10.1107/S1600536814016729/br2240fig3.tif
Représentation des couches disposées parallèlement au plan (100).

**Fig. 4.**
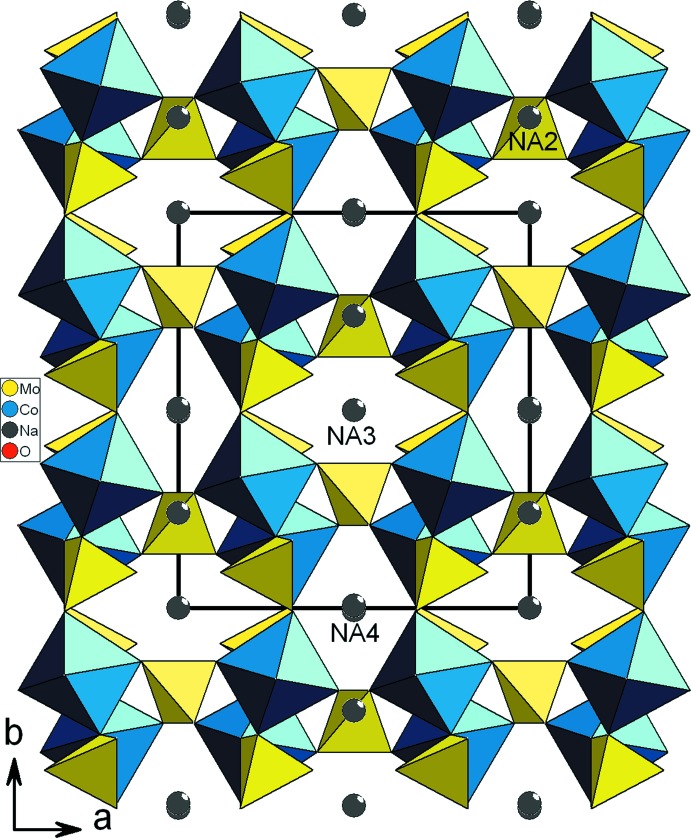
Projection de la structure de Na4Co(MoO4)3 selon c.

4 4 3 c . DOI: 10.1107/S1600536814016729/br2240fig4.tif
Projection de la structure de Na_4_Co(MoO_4_)_3_ selon *c*.

**Fig. 5.**
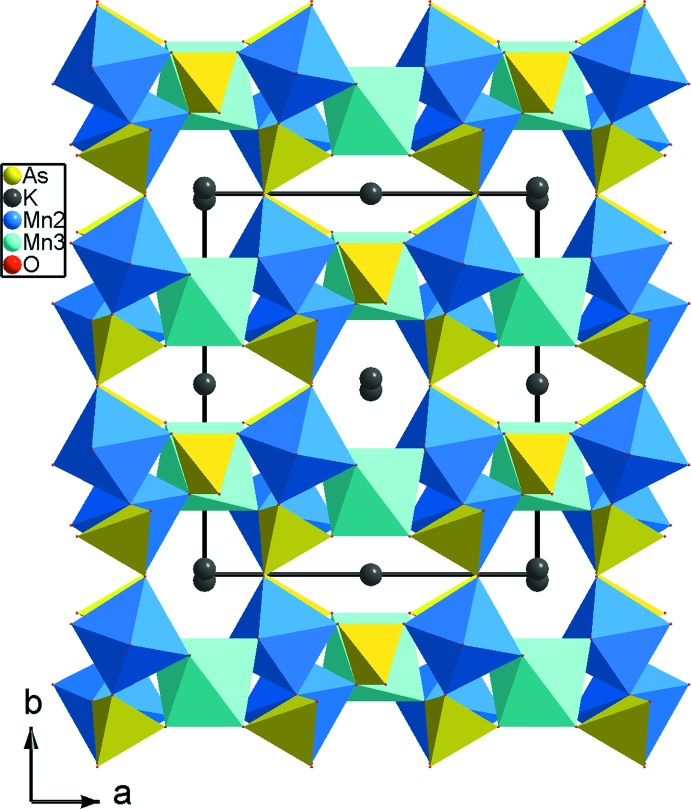
Projection de la structure de K2Mn3(AsO4)3, selon c, montrant la disposition des octaèdres Mn3+O6.

2 3 4 3 c 3+ 6 . DOI: 10.1107/S1600536814016729/br2240fig5.tif
Projection de la structure de K_2_Mn_3_(AsO_4_)_3_, selon *c*, montrant la disposition des octa­èdres Mn^3+^O_6_.

**Fig. 6.**
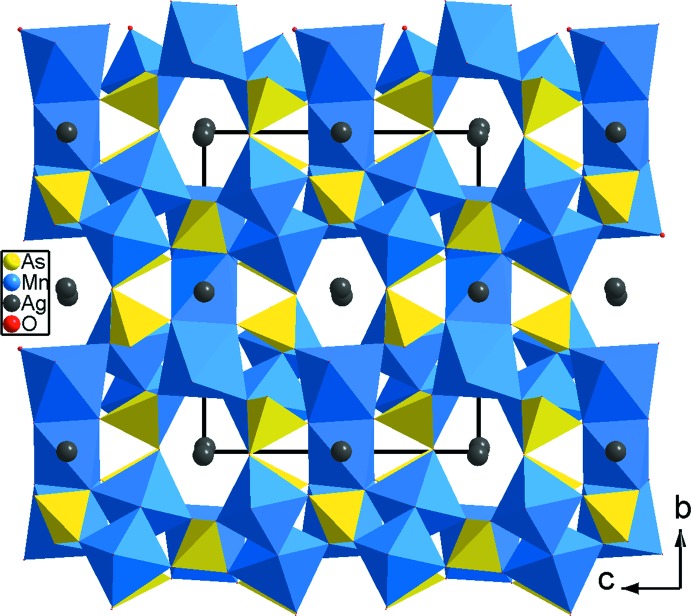
Projection de la structure de Ag1,09Mn3,46(AsO4)3, selon a, montrant la jonction des octaèdres par arêtes.

1,09 3,46 4 3 a . DOI: 10.1107/S1600536814016729/br2240fig6.tif
Projection de la structure de Ag_1,09_Mn_3,46_(AsO_4_)_3_, selon *a*, montrant la jonction des octa­èdes par arêtes.

**Fig. 7.**
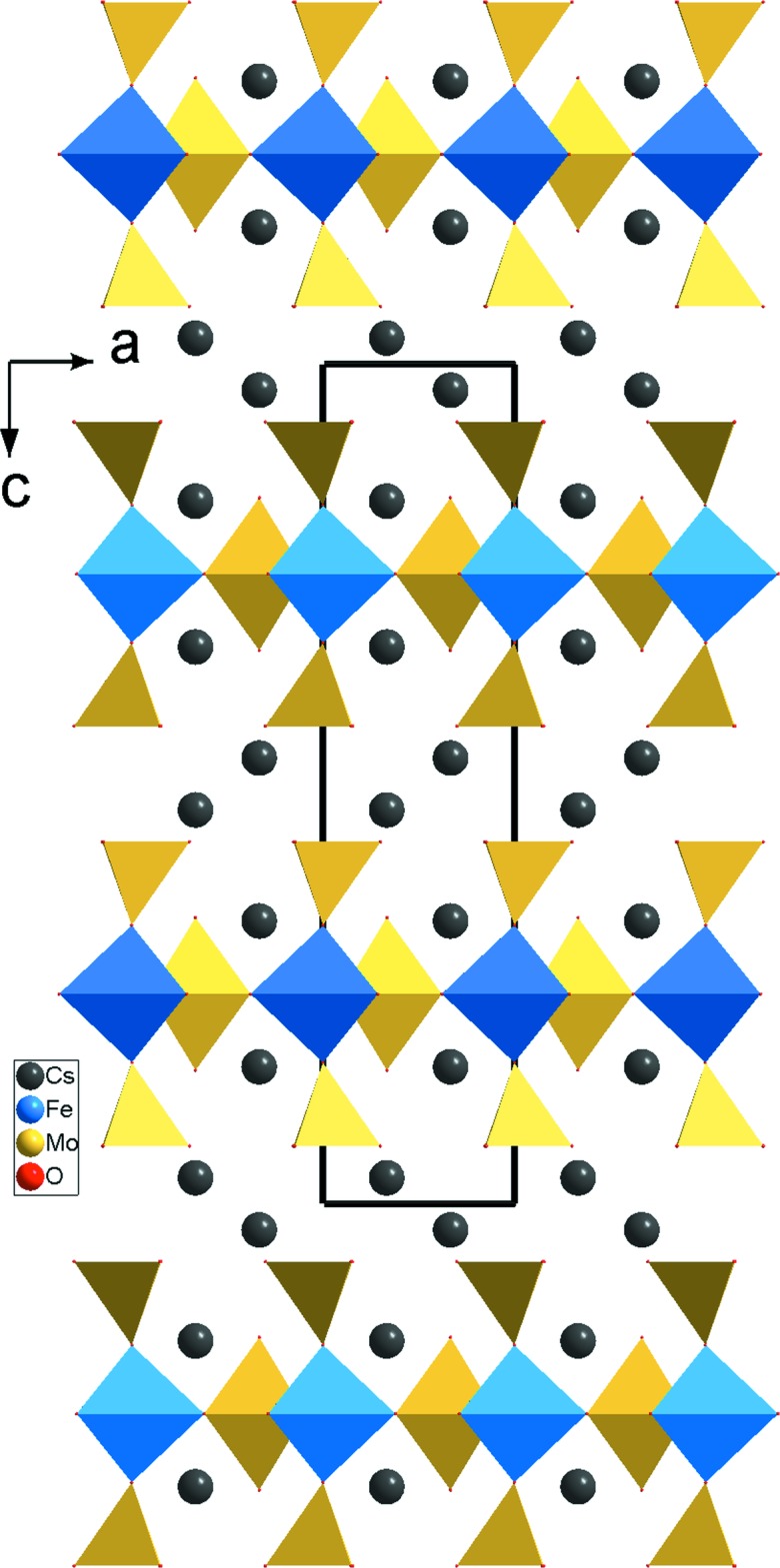
Projection de la structure de Cs4Fe(MoO4)3, selon b, mettant en évidence les espaces inter-couches.

4 4 3 b . DOI: 10.1107/S1600536814016729/br2240fig7.tif
Projection de la structure de Cs_4_Fe(MoO_4_)_3_, selon *b*, mettant en évidence les espaces inter-couches.

CCDC reference: 1015075

Additional supporting information:  crystallographic information; 3D view; checkCIF report

## supplementary crystallographic information

### S1. Comment

Ces dernières années, plusieurs équipes de recherche s'intéressent à l'étude des systèmes quaternaires de type A–M–Mo–O (A = cation monovalent et *M* = métal de transition). En effet, la jonction octaèdres-tétraèdres conduit à des charpentes ouvertes ayant des caractéristiques structurales favorable à la mobilité des ions (Kabbour *et al.*, 2011). De plus, la substitution du métal de transition par un alcalin de petite taille (Li, Na) confère aux matériaux obtenus des propriétés physiques importantes notamment: magnétiques (Namsaraeva *et al.*, 2011; Hidouri *et al.*, 2006), d'insertion et d'extraction (Mikhailova *et al.*, 2010). C'est dans ce cadre, que nous avons choisi l'exploration des systèmes A–Co–Mo–O (A = ion monovalent). Une nouvelle phase de formulation Na_4_Co(MoO_4_)_3_ a été synthétisée par réaction à l'état solide.

Un examen bibliographique montre que le matériau étudié est isostructural aux composés: Na_3_In_2_As_3_O_12_ et Na_3_In_2_P_3_O_12_ (Lii & Ye, 1997) et membre de la famille alluaudite (Moore, 1971; Hatert, 2006; Yakubovich *et al.*, 2005). L'unité *asym*étrique, dans le composé étudié est construite à partir d'un octaèdre CoO_6_ et de deux tétraèdres MoO_4_ connectés par ponts mixtes de type Co–O–Mo (Fig. 1). Dans cette unité les atomes Co1 et Na1 sont situés dans le même site (8f: Wyckoff) avec des taux d'occupation respectivement égaux à 0,503 (5) et 0,497 (6). Alors que les trois autres atomes de sodium (Na2(4e), Na3(4a), Na4(4e)) et l'un des deux atomes de molybdène (Mo1(4e)) se trouvent sur des positions particulières.

Dans la charpente anionique les octaèdres CoO_6_ et les tétraèdres MoO_4_ se lient pour former des chaînes classiques de type CoMoO_8_ avec une disposition en *cis* des tétraèdres Mo2O_4_ (Fig. 2a). Ces dernières se connectent par mize en commun d'arêtes entre les octaèdres CoO_6_ pour donner des rubans de type Co_2_Mo_2_O_14_ (Fig. 2b). Ces rubans se lient au moyen de sommets entre les polyèdres de nature différente pour conduire à des couches disposées parallèlement au plan (100) (Fig. 3). La formation de ce type de couches est la conséquence d'une disposition particulière des dimères Co2O_10_, selon les deux directions [011] et [011]. La jonction entre ces couches est assurée par insertion des tétraèdres Mo1O_4_ et formation de ponts mixtes Co–O–Mo. En effet, chaque tétraèdre Mo1O_4_ partage deux pairs de ses sommets avec respectivement deux couches adjacentes. Par contre dans une couche, chaque tétraèdre Mo2O_4_ partage seulement trois de ses sommets avec les dimères Co2O_10_, le quatrième sommet restant libre forme un groupement molybdyl (*d*(*M*=O)= 1,750 (2) Å) et d'autre part il se dirige vers les canaux où se situent les cations Na3. Cette association conduit à une charpente tridimensionnelle, possédant deux types de canaux larges, à section hexagonale, parallèles à l'axe *c* où logent les cations Na^+^ (Fig. 4).

L'examen des facteurs géométriques dans la structure révèle qu'ils sont conformes à ceux rencontrés dans la littérature (Engel *et al.*, 2005). De plus, le calcul des différentes valences de liaison (BVS), utilisant la formule empirique de Brown (Brown & Altermatt, 1985), conduit aux valeurs des charges des ions suivants: Mo1(5,769), Mo2(5,887), (Co1/Na1)(1,871), Na2(1,116) Na3(0,874), Na4(0,570).

La recherche de structures présentant des aspects communs avec celle de Na_4_Co(MoO_4_)_3_, nous a conduit à la famille des alluaudites de formule générale AA'*M*'*M*_2_(XO_4_)_3_ (A = ion monovalent ou bivalent et *M* = métal de transition) (Moore, 1971). Cependant une diffénce nette est observée d'une part, dans l'arrangement des polyédres et d'autre part dans l'occupation des sites cristallographiques. En effet, dans le composé K_2_Mn_3_(AsO_4_)_3_ (Chaalia *et al.*, 2012), le site (1/2,*y*,3/4) est occupé par l'octaèdre Mn1O_6_ (Fig. 5) par contre dans notre composé, il est occupé par le cation Na2 (Fig. 4).

Une comparaison de la structure avec les formes de type wyllieites et rosemaryites montre qu'ils cristallisent dans le même systéme cristallin monoclinique, et présentent des paramétres de maille similaires, mais ils possédent des groupes d'espace différents: P21/c, P21/n respectivement. Tandisque pour les alluaudites, le groupe d'espace est *C*2/*c*. Pour le composé Ag_1,09_Mn_3,46_(AsO_4_)_3_ de type wyllieite (Frigui *et al.*, 2012), une différence est observée dans la charpente. En effet, les couches sont liées d'une part, par des ponts mixtes Mn—O—As et d'autre part, par partage d'arêtes avec l'octaèdre Mn1O_6_ (Fig. 6). Dans la variété *β*-xenophyllite (Marzouki *et al.*, 2013), le composé Na_4_Li_0.62_Co_5.67_Al_0.71_(AsO_4_)_6_ possède des paramétres de maille, un groupe d'espace (C2/m) et une charpente anionique différente de ceux rencontrés dans notre phase Na_4_Co(MoO_4_)_3_.

Une comparaison de notre structure avec celle des composés ayant une formulation analogue A_4_M(MoO_4_)_3_ (A=Cs, Rb, Na) et (*M*=Fe, Cu, Mn) montre une différence nette d'une part, dans la *sym*étrie cristalline et d'autre part, dans l'arrangement des polyèdres. Les deux composés Cs_4_Fe(MoO_4_)_3_ (Namsaraeva *et al.*, 2011), et Rb_4_Mn(MoO_4_)_3_ (Solodovnikov *et al.*, 1988), sont de *sym*étrie hexagonale P-62*c* et présentent des charpentes bidimensionnelles. En effet, la connection des tétraèdres MoO_4_ aux bipyramides trigonales FeO_5_ engendre des couches disposées parallèlement au plan (001) (Fig. 7). Pour la variété Na_4_Cu(MoO_4_)_3_ (Kelvtsova *et al.*, 1991), elle cristallise dans le systéme triclinique, groupe d'espace P-1. La jonction des différents polyédres conduit aussi à une structure bidimensionnelle. En conclusion, la phase élaboreée Na_4_Co(MoO_4_)_3_ de formulation générale A_4_M(MoO_4_)_3_ est classée, contrairement à ses homologues précedemment cités, une forme Alluaudite.

### S2. Experimental

Les cristaux relatifs à Na_4_Co(MoO_4_)_3_ ont été obtenus par réaction à l'état solide à partir des réactifs: Na_2_CO_3_ (PROLABO, 70128), Co(NO_3_)·6H_2_O (FLUKA, 60832) et (NH4)_2_MoO_4_O_13_ (FLUKA, 69858) pris dans les proportions Na:Co:Mo=3:1:3. Aprés un broyage poussé dans un mortier en agate, le mélange a été mis dans un creuset en porcelaine préchauffé à l'air à 673 K pendant 24 heures en vue d'éliminer les composés volatils. Il est ensuite porté jusqu'à une température de synthèse proche de celle de la fusion à 953 K. Le mélange est abandonné à cette température pendant une semaine pour favoriser la germination et la croissance des cristaux. Par la suite, il a subi en premier lieu un refroidissement lent (5°/jour) jusqu'à 900 K puis rapide (50°/h) jusqu'à la température ambiante. Des cristaux de couleur bleu, de taille suffisante pour les mesures des intensités, ont été séparés du flux par l'eau chaude.

### S3. Refinement

L'affinement des taux d'occupation des atomes de cobalt et de sodium séparément conduit à une formule erronée de type Na_3_Co_1,4_Mo_3_O_12_ où la neutralité électrique n'est pas vérifiée. De plus, La distance moyenne Co1–O égale à 2,19 (1) est supérieure à celle rencontrée dans la bibliographie. En effet, c'est une distance moyenne de type Co/Na–O. L'affinement final a été donc, réalisé en placant les atomes Co1 et Na1 dans le même site, il conduit aux taux d'occupation respectifs égaux à 0,503 (5) et 0,497 (6). La formule finale correspond à une alluaudite de type Na_4_CoMo_3_O_12_. *L*'utilisation des contraintes EADP et EXYZ, autorisées par le programme *SHELXL*-97 (Sheldrick, 2008), pour le couple Co1/Na1 conduit à des ellipsoïdes bien définis. Les densités d'électrons maximum et minimum restants dans la Fourier-différence sont acceptables et sont situées respectivements à 0,88 Å de Mo2 et à 0,51 Å de Na4.

### Crystal data

**Table:**

Na_4_Co(MoO_4_)_3_	*F*(000) = 1172
*M**_r_* = 630.71	*D*_x_ = 3.664 Mg m^−^^3^
Monoclinic, *C*2/*c*	Mo *K*α radiation, λ = 0.71073 Å
Hall symbol: -C 2yc	Cell parameters from 25 reflections
*a* = 12.8770 (8) Å	θ = 10–15°
*b* = 13.4384 (9) Å	µ = 4.85 mm^−^^1^
*c* = 7.1292 (7) Å	*T* = 298 K
β = 112.072 (6)°	Prism, blue
*V* = 1143.27 (15) Å^3^	0.84 × 0.28 × 0.22 mm
*Z* = 4	

### Data collection

**Table:**

Enraf–Nonius CAD-4 diffractometer	1156 reflections with *I* > 2σ(*I*)
Radiation source: fine-focus sealed tube	*R*_int_ = 0.036
Graphite monochromator	θ_max_ = 27.0°, θ_min_ = 2.3°
ω/2θ scans	*h* = −16→16
Absorption correction: ψ scan (North *et al.*, 1968)	*k* = −2→17
*T*_min_ = 0.214, *T*_max_ = 0.344	*l* = −9→9
2898 measured reflections	2 standard reflections every 120 min
1242 independent reflections	intensity decay: 1.4%

### Refinement

**Table:**

Refinement on *F*^2^	Primary atom site location: structure-invariant direct methods
Least-squares matrix: full	Secondary atom site location: difference Fourier map
*R*[*F*^2^ > 2σ(*F*^2^)] = 0.025	*w* = 1/[σ^2^(*F*_o_^2^) + (0.0303*P*)^2^ + 3.9296*P*] where *P* = (*F*_o_^2^ + 2*F*_c_^2^)/3
*wR*(*F*^2^) = 0.065	(Δ/σ)_max_ < 0.001
*S* = 1.12	Δρ_max_ = 1.14 e Å^−^^3^
1242 reflections	Δρ_min_ = −0.81 e Å^−^^3^
95 parameters	Extinction correction: *SHELXL97* (Sheldrick, 2008), Fc^*^=kFc[1+0.001xFc^2^λ^3^/sin(2θ)]^-1/4^
0 restraints	Extinction coefficient: 0.0136 (5)

### Special details

**Table:**

Geometry. All e.s.d.'s (except the e.s.d. in the dihedral angle between two l.s. planes) are estimated using the full covariance matrix. The cell e.s.d.'s are taken into account individually in the estimation of e.s.d.'s in distances, angles and torsion angles; correlations between e.s.d.'s in cell parameters are only used when they are defined by crystal symmetry. An approximate (isotropic) treatment of cell e.s.d.'s is used for estimating e.s.d.'s involving l.s. planes.
Refinement. Refinement of *F*^2^ against ALL reflections. The weighted *R*-factor *wR* and goodness of fit *S* are based on *F*^2^, conventional *R*-factors *R* are based on *F*, with *F* set to zero for negative *F*^2^. The threshold expression of *F*^2^ > σ(*F*^2^) is used only for calculating *R*-factors(gt) *etc*. and is not relevant to the choice of reflections for refinement. *R*-factors based on *F*^2^ are statistically about twice as large as those based on *F*, and *R*- factors based on ALL data will be even larger.

### Fractional atomic coordinates and isotropic or equivalent isotropic displacement parameters (Å^2^)

**Table:**

	*x*	*y*	*z*	*U*_iso_*/*U*_eq_	Occ. (<1)
Mo1	0.0000	0.21721 (3)	0.2500	0.01688 (16)	
Mo2	0.26106 (3)	0.89054 (2)	0.37349 (4)	0.01710 (15)	
Co1	0.28417 (6)	0.16208 (6)	0.37580 (10)	0.01443 (19)	0.503 (5)
Na1	0.28417 (6)	0.16208 (6)	0.37580 (10)	0.01443 (19)	0.497 (6)
Na2	0.0000	0.23993 (19)	0.7500	0.0245 (5)	
Na3	0.0000	0.0000	0.0000	0.0372 (6)	
Na4	0.5000	0.0060 (3)	0.7500	0.0454 (7)	
O1	0.2761 (3)	0.8197 (2)	0.1715 (4)	0.0283 (7)	
O2	0.3244 (3)	0.8298 (2)	0.6100 (4)	0.0264 (6)	
O3	0.1067 (3)	0.1352 (2)	0.2466 (5)	0.0324 (7)	
O4	0.3248 (3)	0.0080 (3)	0.3900 (5)	0.0345 (7)	
O5	0.1179 (3)	0.9099 (2)	0.3156 (5)	0.0290 (7)	
O6	0.0439 (2)	0.2916 (2)	0.4718 (4)	0.0238 (6)	

### Atomic displacement parameters (Å^2^)

**Table:**

	*U*^11^	*U*^22^	*U*^33^	*U*^12^	*U*^13^	*U*^23^
Mo1	0.0256 (3)	0.0134 (2)	0.0097 (2)	0.000	0.00436 (17)	0.000
Mo2	0.0212 (2)	0.0184 (2)	0.0106 (2)	−0.00135 (12)	0.00467 (13)	0.00017 (11)
Co1	0.0183 (4)	0.0159 (4)	0.0092 (3)	0.0006 (3)	0.0054 (3)	−0.0009 (3)
Na1	0.0183 (4)	0.0159 (4)	0.0092 (3)	0.0006 (3)	0.0054 (3)	−0.0009 (3)
Na2	0.0248 (11)	0.0317 (12)	0.0208 (11)	0.000	0.0131 (9)	0.000
Na3	0.0506 (16)	0.0234 (12)	0.0239 (13)	0.0014 (12)	−0.0018 (11)	−0.0011 (11)
Na4	0.0233 (12)	0.0505 (18)	0.0527 (19)	0.000	0.0031 (12)	0.000
O1	0.0368 (16)	0.0338 (17)	0.0165 (13)	−0.0011 (14)	0.0124 (12)	−0.0011 (12)
O2	0.0339 (15)	0.0267 (15)	0.0142 (13)	0.0072 (13)	0.0041 (11)	0.0009 (11)
O3	0.0363 (16)	0.0248 (15)	0.0289 (16)	0.0052 (13)	0.0042 (13)	−0.0075 (13)
O4	0.0416 (18)	0.0309 (17)	0.0294 (16)	−0.0123 (15)	0.0114 (14)	0.0030 (14)
O5	0.0266 (15)	0.0268 (15)	0.0329 (16)	0.0042 (12)	0.0104 (13)	0.0043 (13)
O6	0.0306 (15)	0.0293 (15)	0.0127 (12)	−0.0020 (12)	0.0094 (11)	−0.0047 (11)

### Geometric parameters (Å, º)

**Table:**

Mo1—O3^i^	1.769 (3)	Na2—O6	2.361 (3)
Mo1—O3	1.769 (3)	Na2—O2^viii^	2.424 (3)
Mo1—O6	1.774 (3)	Na2—O2^ix^	2.424 (3)
Mo1—O6^i^	1.774 (3)	Na2—O5^x^	2.458 (4)
Mo2—O5	1.750 (3)	Na2—O5^v^	2.458 (4)
Mo2—O4^ii^	1.762 (3)	Na3—O5^xi^	2.503 (3)
Mo2—O2	1.772 (3)	Na3—O5^xii^	2.503 (3)
Mo2—O1	1.796 (3)	Na3—O3^xiii^	2.543 (3)
Co1—O4	2.129 (4)	Na3—O3	2.543 (3)
Co1—O2^iii^	2.146 (3)	Na3—O5^iii^	2.646 (3)
Co1—O3	2.149 (3)	Na3—O5^xiv^	2.646 (3)
Co1—O6^iv^	2.159 (3)	Na4—O4^xv^	2.706 (3)
Co1—O1^v^	2.164 (3)	Na4—O4	2.706 (3)
Co1—O1^vi^	2.237 (3)	Na4—O4^xvi^	2.795 (4)
Na2—O6^vii^	2.361 (3)	Na4—O4^xvii^	2.795 (4)
			
O3^i^—Mo1—O3	102.9 (2)	O2^iii^—Co1—O3	101.57 (12)
O3^i^—Mo1—O6	109.14 (14)	O4—Co1—O6^iv^	93.97 (13)
O3—Mo1—O6	111.98 (14)	O2^iii^—Co1—O6^iv^	83.55 (11)
O3^i^—Mo1—O6^i^	111.98 (14)	O3—Co1—O6^iv^	171.19 (12)
O3—Mo1—O6^i^	109.14 (14)	O4—Co1—O1^v^	99.48 (13)
O6—Mo1—O6^i^	111.42 (19)	O2^iii^—Co1—O1^v^	166.02 (13)
O5—Mo2—O4^ii^	107.81 (16)	O3—Co1—O1^v^	90.22 (12)
O5—Mo2—O2	111.10 (15)	O6^iv^—Co1—O1^v^	83.85 (11)
O4^ii^—Mo2—O2	108.20 (15)	O4—Co1—O1^vi^	173.29 (12)
O5—Mo2—O1	108.10 (15)	O2^iii^—Co1—O1^vi^	90.23 (11)
O4^ii^—Mo2—O1	109.90 (15)	O3—Co1—O1^vi^	80.90 (12)
O2—Mo2—O1	111.66 (14)	O6^iv^—Co1—O1^vi^	92.00 (12)
O4—Co1—O2^iii^	87.41 (13)	O1^v^—Co1—O1^vi^	84.19 (12)
O4—Co1—O3	93.43 (13)		

Symmetry codes: (i) −*x*, *y*, −*z*+1/2; (ii) *x*, *y*+1, *z*; (iii) *x*, −*y*+1, *z*−1/2; (iv) −*x*+1/2, −*y*+1/2, −*z*+1; (v) *x*, −*y*+1, *z*+1/2; (vi) −*x*+1/2, *y*−1/2, −*z*+1/2; (vii) −*x*, *y*, −*z*+3/2; (viii) −*x*+1/2, *y*−1/2, −*z*+3/2; (ix) *x*−1/2, *y*−1/2, *z*; (x) −*x*, −*y*+1, −*z*+1; (xi) −*x*, −*y*+1, −*z*; (xii) *x*, *y*−1, *z*; (xiii) −*x*, −*y*, −*z*; (xiv) −*x*, *y*−1, −*z*+1/2; (xv) −*x*+1, *y*, −*z*+3/2; (xvi) −*x*+1, −*y*, −*z*+1; (xvii) *x*, −*y*, *z*+1/2.
