# Supplementary figures and images for: Crystal structure of alluaudite-type Na4Co(MoO4)3
Source: Acta Crystallogr Sect E Struct Rep Online. 2014 Aug 1;70(Pt 9):i47–8. doi: 10.1107/S1600536814016729 (PMC4186203; doi:10.1107/S1600536814016729)

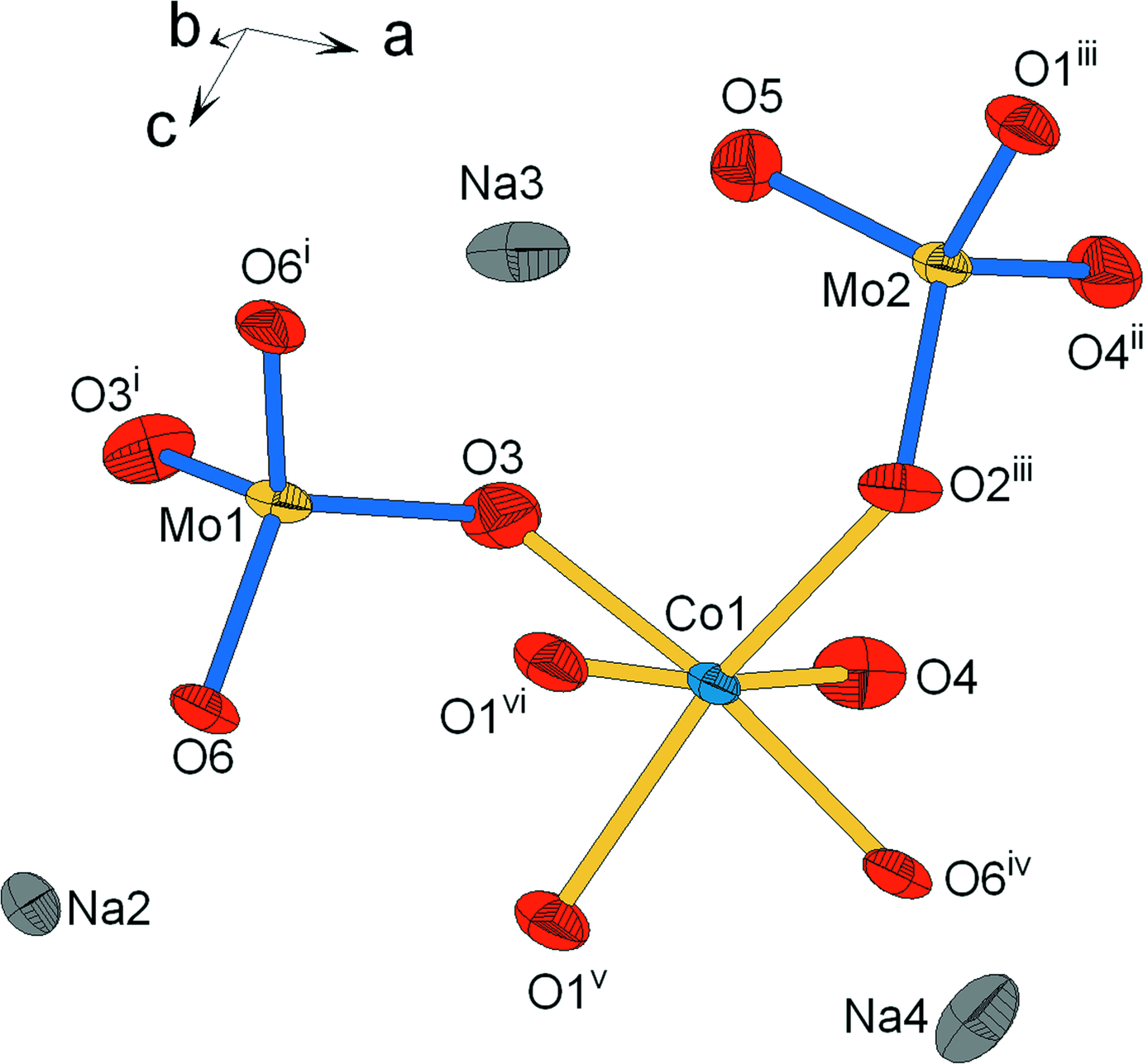

Supplement: Supplementary file 3 [file e-70-00i47-fig1.tif]

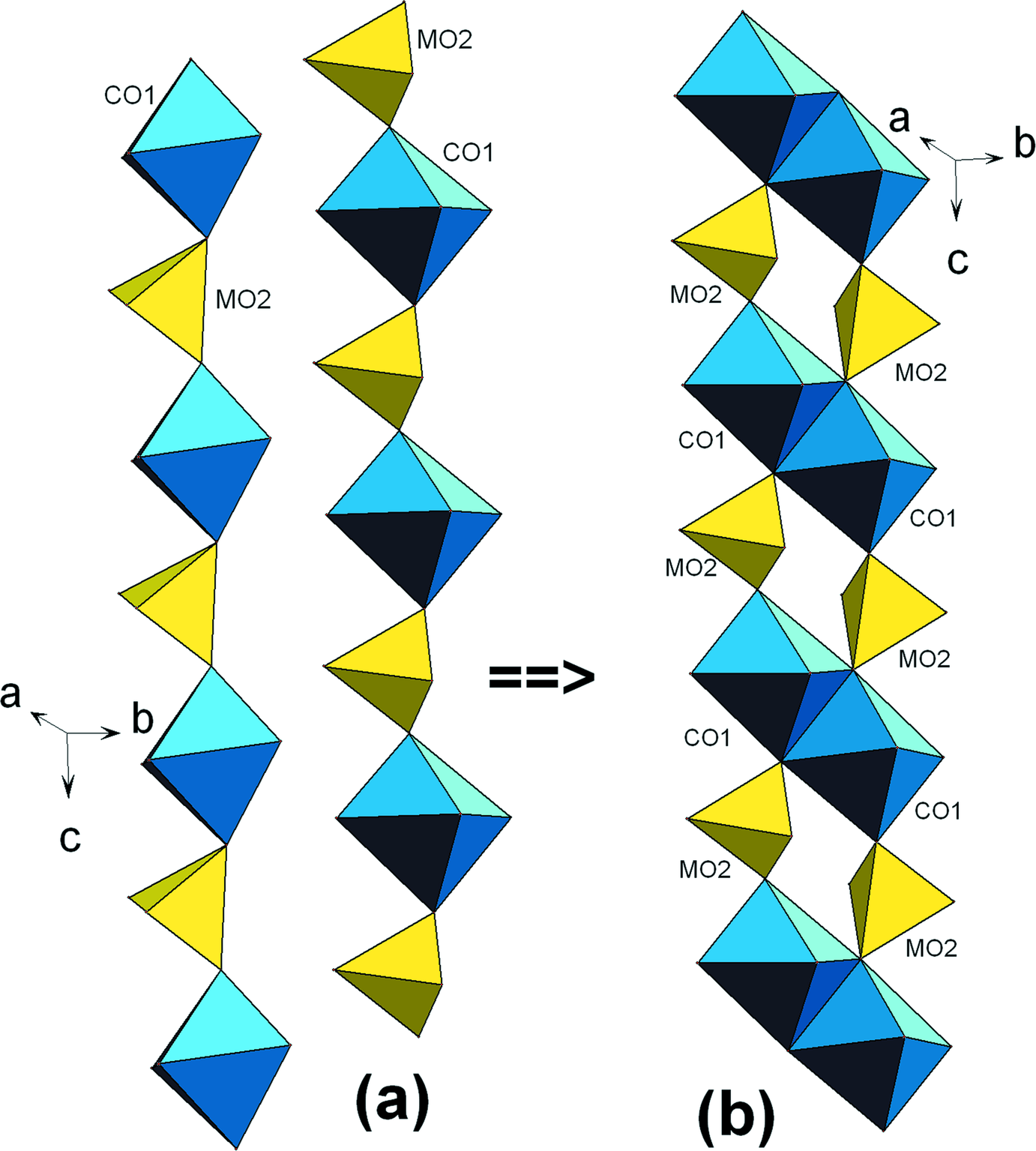

Supplement: Supplementary file 4 [file e-70-00i47-fig2.tif]

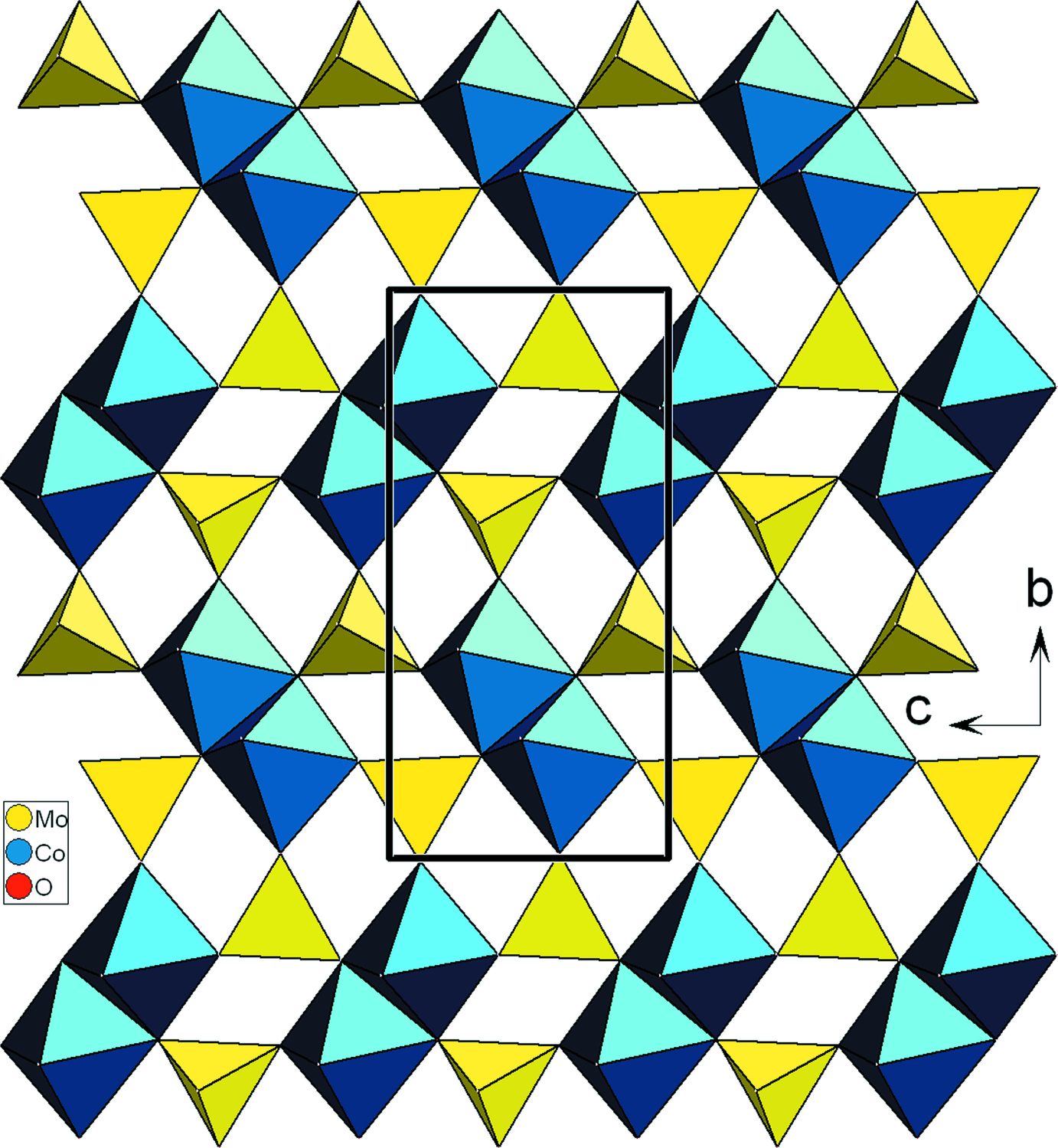

Supplement: Supplementary file 5 [file e-70-00i47-fig3.tif]

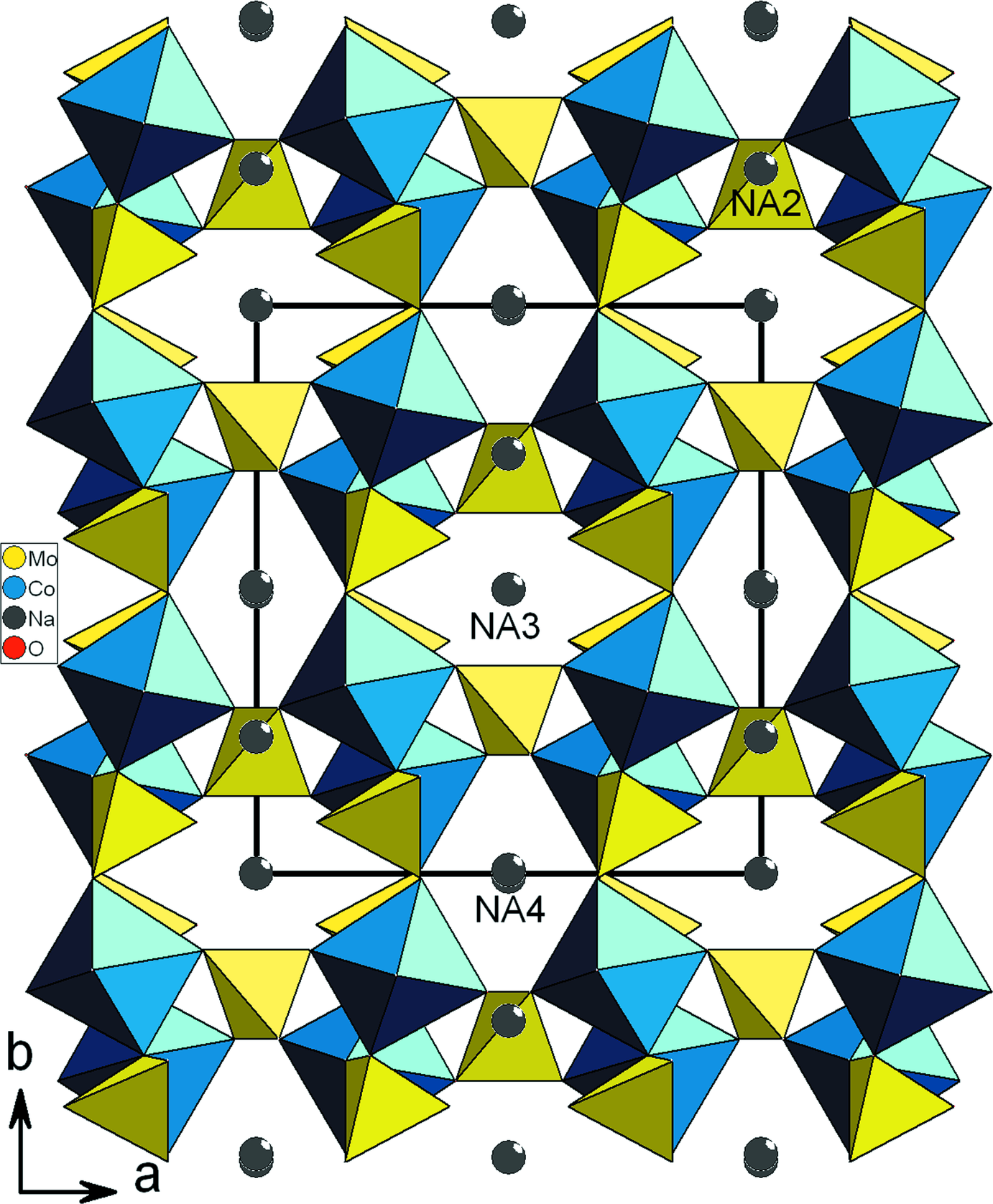

Supplement: Supplementary file 6 [file e-70-00i47-fig4.tif]

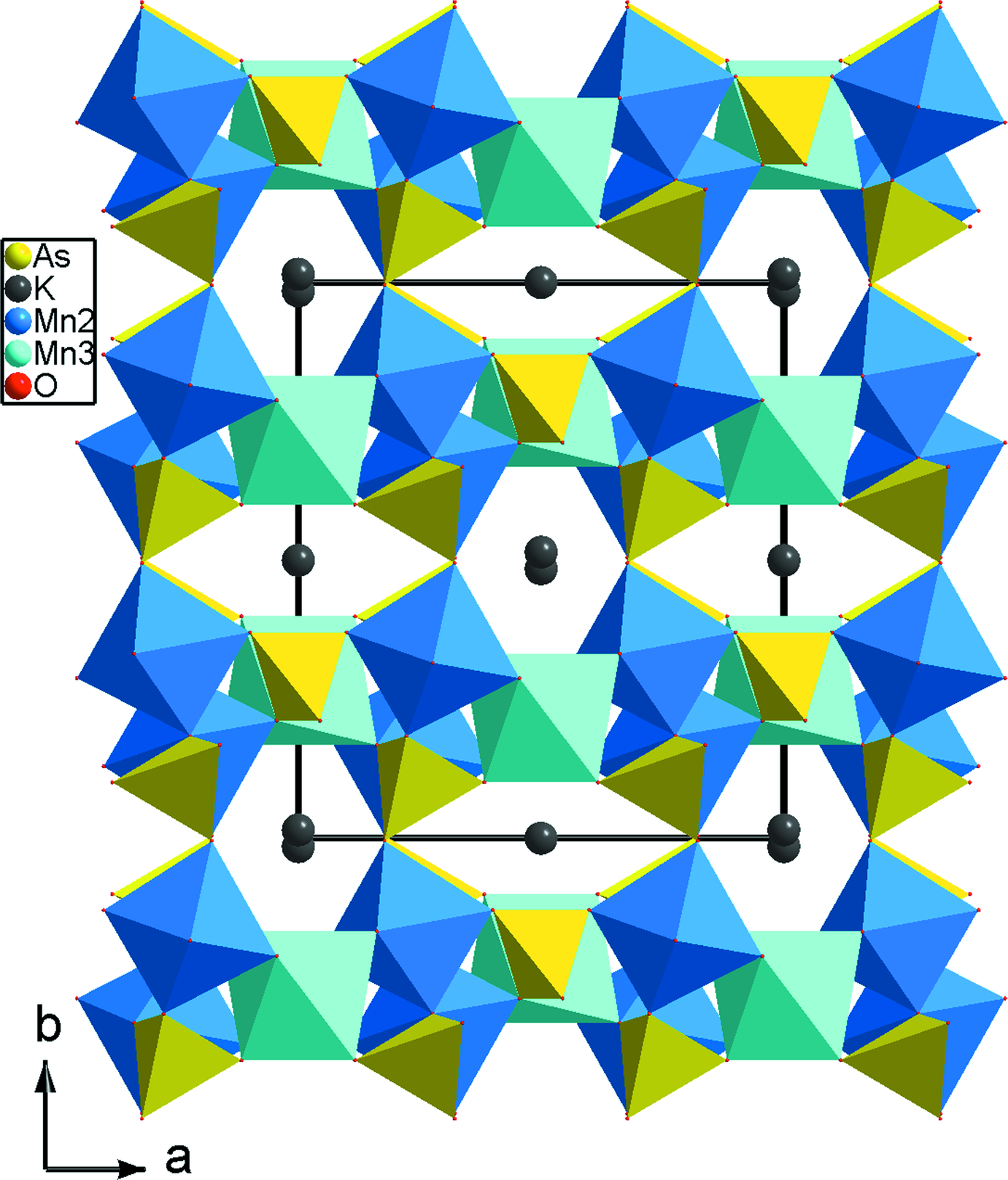

Supplement: Supplementary file 7 [file e-70-00i47-fig5.tif]

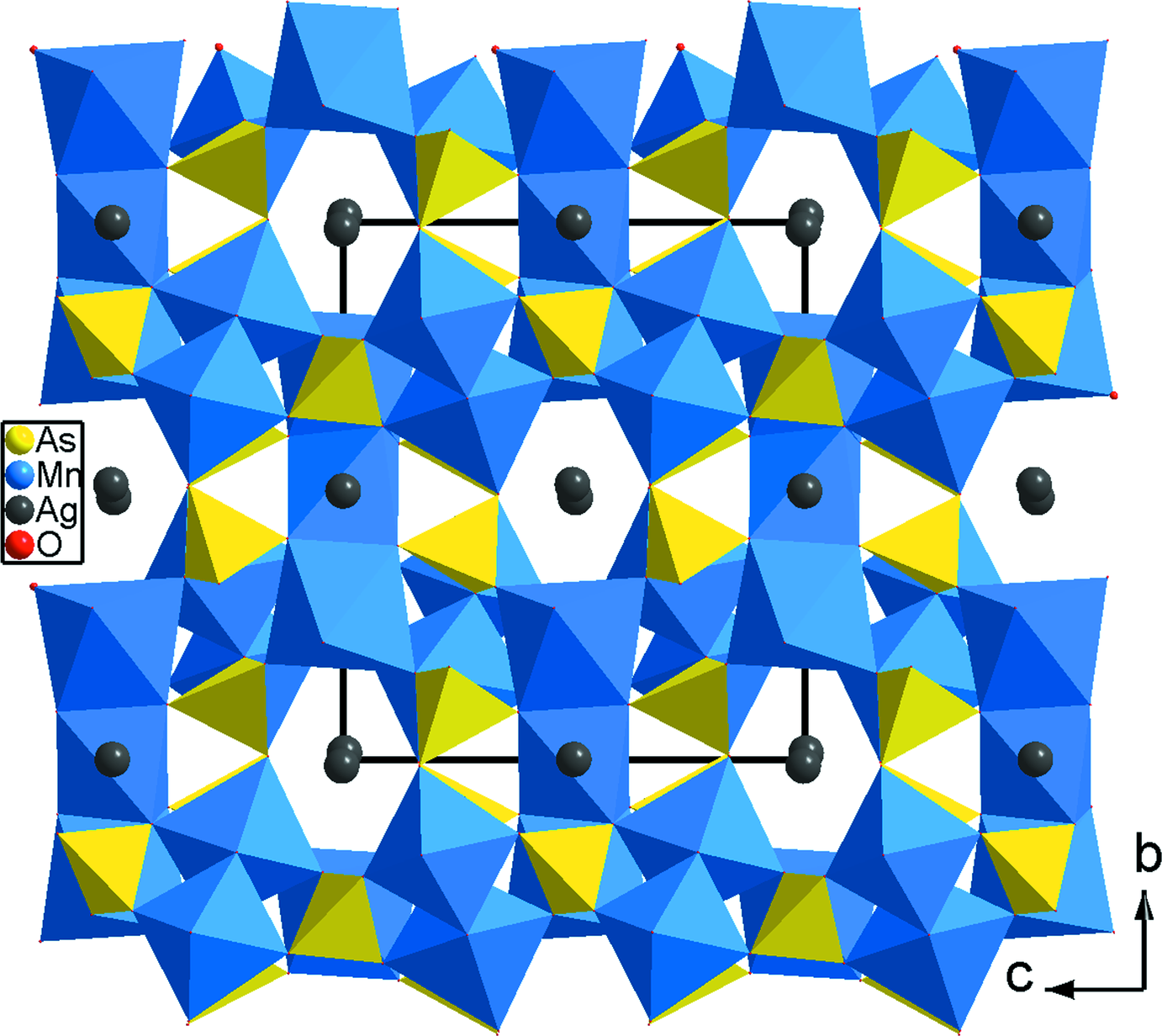

Supplement: Supplementary file 8 [file e-70-00i47-fig6.tif]

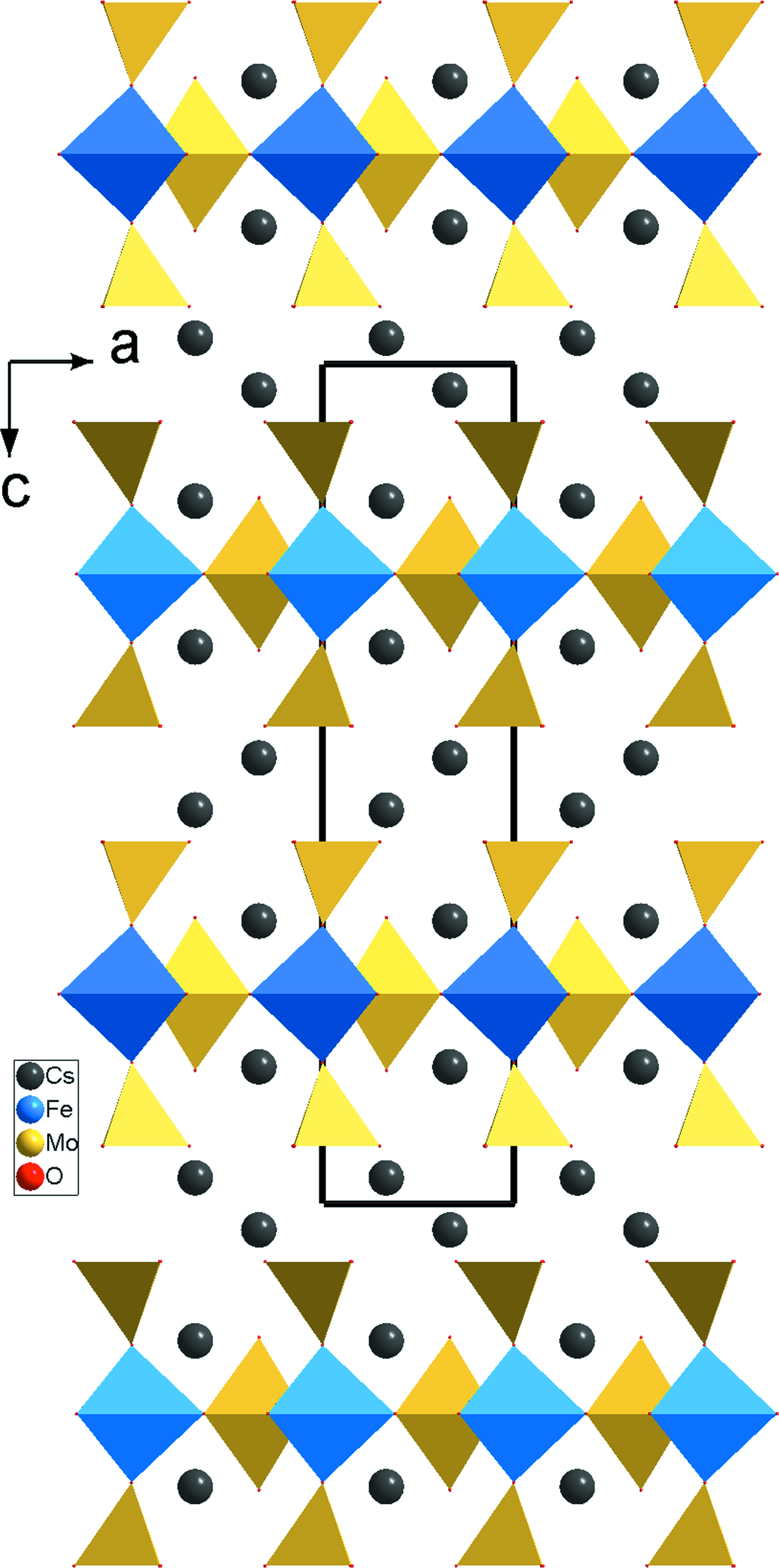

Supplement: Supplementary file 9 [file e-70-00i47-fig7.tif]
